# Supplementary material for: Independent domestication and cultivation histories of two West African indigenous fonio millet crops
Source: Nat Commun. 2025 Apr 30;16:4067. doi: 10.1038/s41467-025-59454-2 (PMC12044004; doi:10.1038/s41467-025-59454-2)
Supplement: Supplementary file 1 — Supplementary Information [file 41467_2025_59454_MOESM1_ESM.pdf]

Independent domestication and cultivation histories of two West African indigenous fonio millet crops

Kaczmarek *et al.*

Supplementary Note 1. Structure obtained with sNMF on the k-mer dataset

The analysis performed with the k-mer dataset led to similar results (Supplementary Fig. 9, Supplementary Fig. 10, Supplementary Fig. 11). The cross-entropy decreased strongly from K=2 to K=6, reaching a minimum at K=11 (Supplementary Fig. 9). We observed only minor differences, particularly in the order of appearance of the clusters (Supplementary Fig. 10). At K=3, *D. ternata* individuals grouped together and differed from a group of *D. iburua* and a group of *D. exilis* and *D. longiflora*. The same geographic structure pattern of *D. exilis* obtained with the SNP dataset was highlighted from K=4. At K=5, *D. longiflora* individuals differed from *D. exilis* and intraspecific clusters of *D. exilis* were captured from K=6. The splitting of *D. iburua* and *D. longiflora*, each into two groups, as seen in the PCA (Supplementary Fig. 6), occurred at K=9 and K=10, respectively (Supplementary Fig. 10). The maps of genetic clusters inferred with both datasets showed a highly consistent clustering of individuals when using K=6 for the k-mer dataset and K=8 for the SNP dataset (Supplementary Fig. 11). These results supported the use of the SNP dataset (with a 5% threshold) to analyse the interspecific evolutionary history of the fonio millet species complex in subsequent analyses.

Supplementary Method 1. DNA extraction from herbarium leaves

- prepare 2x CTAB isolation buffer:
    - 100mM Tris-HCl pH8
    - 1.4M NaCl
    - 20 mM EDTA
    - 2% CTAB
    - 0.2% 2-mercaptoethanol
    - + 2% of sodium bisulphite (added at the extraction time)
- grind 0.01-0.05 g of leaf
- add 800 µL of pre-heated extraction buffer (65°C)
- incubate at 65°C for 60 minutes
- add 800 µL of Chloroform Isoamyl-alcohol CIAA (24 :1), mix well
- centrifuge at 8000 rpm for 10 minutes
- add RNAse A in order to obtain final concentration at 1 µg/mL
- incubate 30 minutes at 37°C
- transfer aqueous phase to new tube
- add 550 µL (2/3 vol) of cold Isopropanol, mix gently
- precipitate 5 days at -20°C
- centrifuge at 5000 rpm for 10 minutes
- discard supernatant
- add 600 µL of wash buffer (76% Ethanol, 10mM Ammonium acetate), shake gently to resuspend pellet
- incubate for 20 minutes at RT
- centrifuge at 5000 rpm for 10 minutes

- discard supernatant, allow to dry at RT
- add 50 µL of TE buffer (10mM Tris-HCL pH7.4, 1mM EDTA, pH8.0)

Supplementary Method 2. Twisst pipeline

We used the Twisst pipeline (https://github.com/simonhmartin/twisst?tab=readme-ov-file) to determine which tree topology was best supported by our data. Prior to analysis, we phased the haplotypes with beagle v4.0. We ran Twisst with the *phyml_sliding_windows.py* script which generates neighbour joining trees for windows of 50 SNPs using PhyML v3.0. To account for the difference in population size between the two cultivated species, we implemented this method with a reduced sample of 21 *D. exilis* representative of the species clustering pattern and geographic distribution. For the other three species, we included individuals with membership coefficients > 0.6. We ran Twisst either with six populations (the two *D. exilis* populations were merged), or with four populations corresponding to the four species and by deleting the two more distinct wild relative populations. No root is considered when computing the weights of each sub-trees but rooting was performed afterwards using the root function from the ape R package, in which case the *D. ternata* population from Côte d'Ivoire was specified as the outgroup when six populations were used. We used the ape v.5.7-1 R package and the twisst plotting functions to display results and best weighted tree topologies.

Supplementary Table 1. Summary of the passport information for the four species analysed in this study. We distinguished between the projects that led to the sampling of material stored in national collections and duplicated in the ARCAD genebank, the origin of the vegetative material used for sequencing, and the origin of the sequences used in the study.

Species	Statute	Project(s)	Samples collected from	Source of the sequences produced	Number of accessions
Digitaria exilis	Cultivated	Fao-IRD	IRD	Abrouk et al. 2020	157
		Africrop waap-fonio	ARCAD	This study	46
D. iburua		Africrop waap-fonio	ARCAD	This study	26
D. longiflora	Wild	NA	MNHN, CIRAD, IFAN and NBC herbariums	Abrouk et al. 2020	14
D. ternata			MNHN herbarium	This study	16
			CIRAD herbarium	This study	6

MNHN = Muséum d'Histoire Naturelle, Paris, France; NBC = Naturalis Biodiversity Center, Leiden, The Netherlands; IFAN = Institut Fondamental d'Afrique Noire, Dakar, Senegal.

Supplementary Table 2. Statistical comparisons of genetic diversity statistics for each pair of populations. Two-tailed Welch *t*-test were performed.

Diversity statistic	Species pair	*t*-statistics	Degrees of freedom	*p*-value
π	*D. exilis / D. longiflora*	-802.11	105811	< 2.2e-16
	D. exilis / D. iburua	-484.13	99379	< 2.2e-16
	D. exilis / D. ternata	-630.54	87051	< 2.2e-16
	D. longiflora / D. iburua	224.33	124424	< 2.2e-16
	D. longiflora / D. ternata	-2.0232	114096	0.04305
	D. iburua / D. ternata	-191.78	119035	< 2.2e-16
ϴ	*D. exilis / D. longiflora*	-826.25	106982	< 2.2e-16
	D. exilis / D. iburua	-369.8	121511	< 2.2e-16
	D. exilis / D. ternata	-622.12	95239	< 2.2e-16
	D. longiflora / D. iburua	483.57	118052	< 2.2e-16
	D. longiflora / D. ternata	90.247	121295	< 2.2e-16
	D. iburua / D. ternata	-331.74	106559	< 2.2e-16
Jaccard index (k-mers)	*D. exilis / D. longiflora*	-30.484	183.84	< 2.2e-16
	D. exilis / D. iburua	-35.162	182.72	< 2.2e-16
	D. exilis / D. ternata	-30.188	154.61	< 2.2e-16
	D. longiflora / D. iburua	-13.511	155.26	< 2.2e-16
	D. longiflora / D. ternata	-12.341	132.41	< 2.2e-16
	D. iburua / D. ternata	-1.4552	183.22	0.1473

Supplementary Table 3. Genetic diversity estimates computed for each species with SNP datasets, applying a filter of 20% for the locus missing rate, for each species separately. Statistics were computed using sliding windows of size 50kb and step sizes of 10kb. Mean values with standard deviation are shown. Source data are provided as a Source Data file.

	D. exilis	***D. longiflora***	***D. iburua***	***D. ternata***
#ind	199	14	21	11
#sites	15,257,883	9,693,764	2,590,230	1,546,299
π	0.019 (SD = 0.023)	0.155 (SD=0.036)	0.107 (SD=0.040)	0.155 (SD=0.49)
ϴ	0.033 (SD=0.021)	0.164 (SD=0.034)	0.083 (SD=0.026)	0.146 (SD=0.040)
Tajima's D	-1.44 (SD = 1.11)	-0.21 (SD=0.42)	0.98 (SD=0.77)	0.26 (SD=0.52)
S	3,151,567 (20.66%)	5,946,094 (61.34%)	951,947 (36.75%)	836,109 (54.07%)

#ind = Number of considered individuals; #sites = Total number of sites across the genome with a locus missing rate < 20% for the species considered; π = Nucleotide diversity; ϴ = Watterson's estimator of nucleotide diversity; S = Total number (and proportion) of polymorphic sites across the genome with a locus missing rate < 20%.

Supplementary Table 4. Pairwise population distances computed between the seven genetic clusters inferred with sNMF. Distances were computed with the SNP dataset filtered at 5% for locus missingness, without filtering for minor allele frequency (1,919,119 SNPs). The lower diagonal represents the pairwise nucleotide divergence (D_{xy}) and the upper diagonal represents the net pairwise distance (D_a).

**Group**	***D. exilis* (Guinea)**	***D. exilis* (north)**	***D. longiflora* (west)**	***D. longiflora* (east)**	***D. iburua***	***D. ternata***	***D. ternata* (Côte d'Ivoire)**
***D. exilis* (Guinea)**	-	0.0006	0.008	0.05	0.16	0.14	0.14
***D. exilis* (north)**	0.005	-	0.008	0.05	0.16	0.14	0.14
***D. longiflora* (west)**	0.02	0.02	-	0.04	0.16	0.14	0.14
***D. longiflora* (east)**	0.10	0.10	0.11	-	0.17	0.15	0.15
D. iburua	0.22	0.22	0.23	0.27	-	0.02	0.11
D. ternata	0.19	0.19	0.21	0.25	0.13	-	0.10
***D. ternata* (Côte d'Ivoire)**	0.20	0.20	0.22	0.26	0.22	0.21	-

Supplementary Table 5. Parameter estimates of the best scenario inferred with fastsimcoal (m01 with bottlenecks), computed with parametric bootstraps using 100 independent pseudo-observed datasets of 200,000 non-recombining DNA segments of 1000 bp.

Parameter	Minimum	Mean	Maximum
N_{exilis}	83,770	83,785	83,795
N_{iburua}	43,219	57,528	71,949
$N_{longiflora}$	292,392	292,392	292,392
$N_{ternata}$	719,184	719,184	719,184
$N_{ancestral}$	1,285,314	1,426,905	1,479,447
$T_{long\text{-}exi}$	23,858	25,978	29,326
$T_{tern\text{-}ibu}$	8,810	9,932	11,650
T_{ANC}	764,065	776,807	787,013

Supplementary Table 6. Prior distributions used for coalescent simulations for each model, in order to infer the best scenario of divergence among the four *Digitaria* species.

Model(s)	Parameter	Distribution	Lower bound	Upper bound
m01 m02 m03 m04 m05 m06	N_{exilis}	log uniform	1,000	100,000
	N_{iburua}	log uniform	1,000	100,000
	$N_{longiflora}$	log uniform	10,000	1,000,000
	$N_{ternata}$	log uniform	10,000	1,000,000
	$N_{ancestral}$	log uniform	100,000	2,000,000
m01	$T_{long\text{-}exi}$	uniform	1,000	50,000
	$T_{tern\text{-}ibu}$	uniform	1,000	50,000
	T_{ANC}	uniform	$T_{long\text{-}exi}$	1,000,000
m02	$T_{long\text{-}exi}$	uniform	1,000	50,000
	$T_{tern\text{-}ibu}$	uniform	1,000	50,000
	T_{ANC}	uniform	$T_{tern\text{-}ibu}$	100,0000
m03 m06	$T_{long\text{-}exi}$	uniform	1,000	50,000
	T_{ANC}	uniform	$T_{long\text{-}exi}$	100,0000
	$T_{tern\text{-}ibu}$	uniform	$T_{long\text{-}exi}$	T_{ANC}
m04 m05	$T_{tern\text{-}ibu}$	uniform	1,000	50,000
	T_{ANC}	uniform	$T_{tern\text{-}ibu}$	1,000,000
	$T_{long\text{-}exi}$	uniform	$T_{tern\text{-}ibu}$	T_{ANC}

Prior distributions are uniform or log-uniform. Bounded values represent initial search ranges for parameter estimation. Parameters are systematically bounded by the lower value, with no restriction of overpassing the upper value, except when the parameter is bounded by another parameter. N_X represent effective population size of population X, and $T_{X\text{-}Y}$ represent the divergence time in generation between population X and population Y.

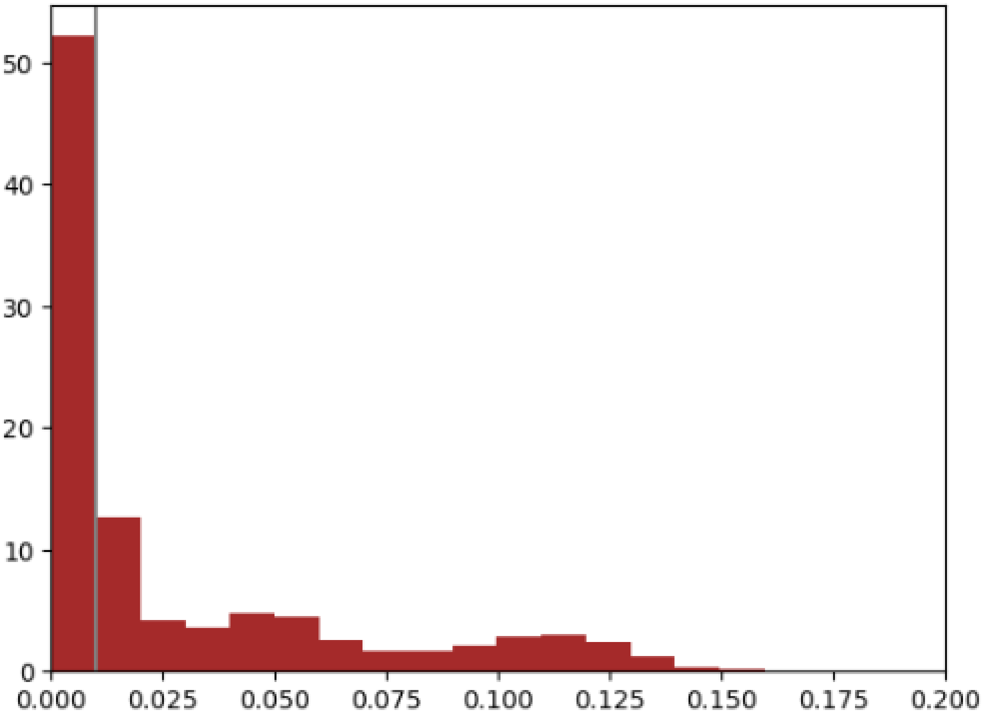

Supplementary Fig. 1. **Minor allele frequency for the SNP dataset filtered at 5% for locus missingness (1,910,119 SNPs, 247 individuals).**

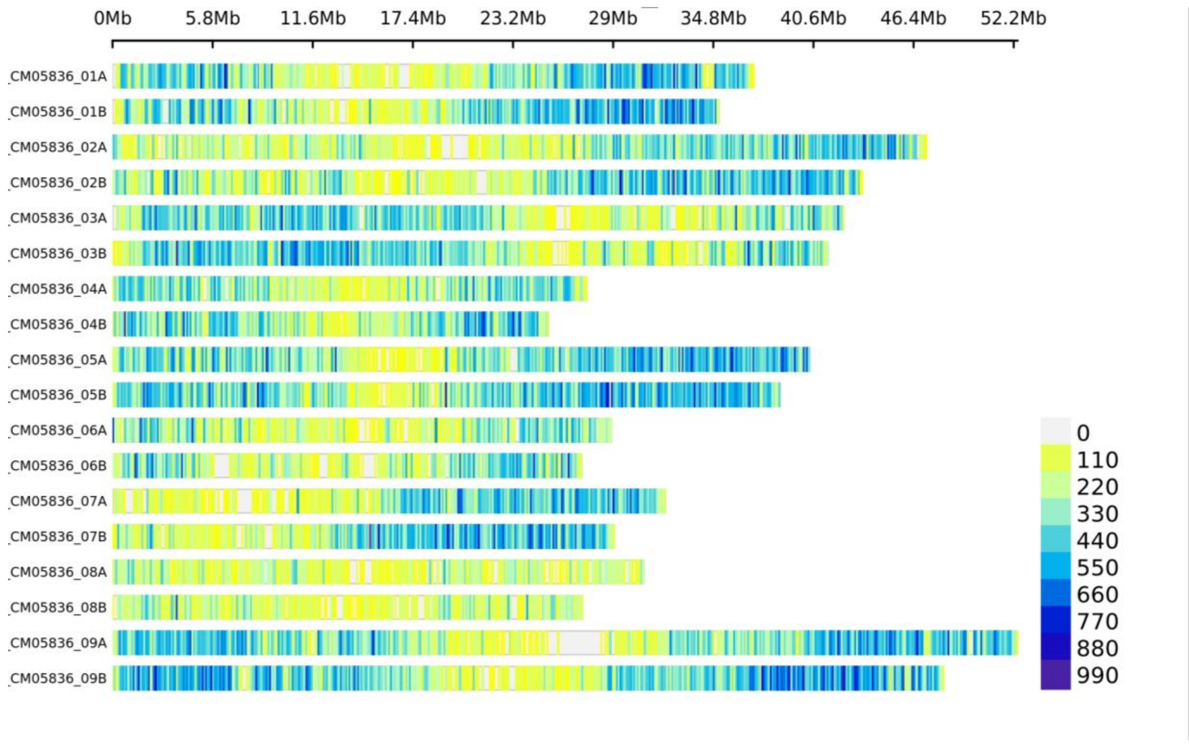

Supplementary Fig. 2. SNP density across the 18 *D. exilis* chromosomes computed with the dataset of 1,910,911 SNPs, and with bin sizes of 100kb.

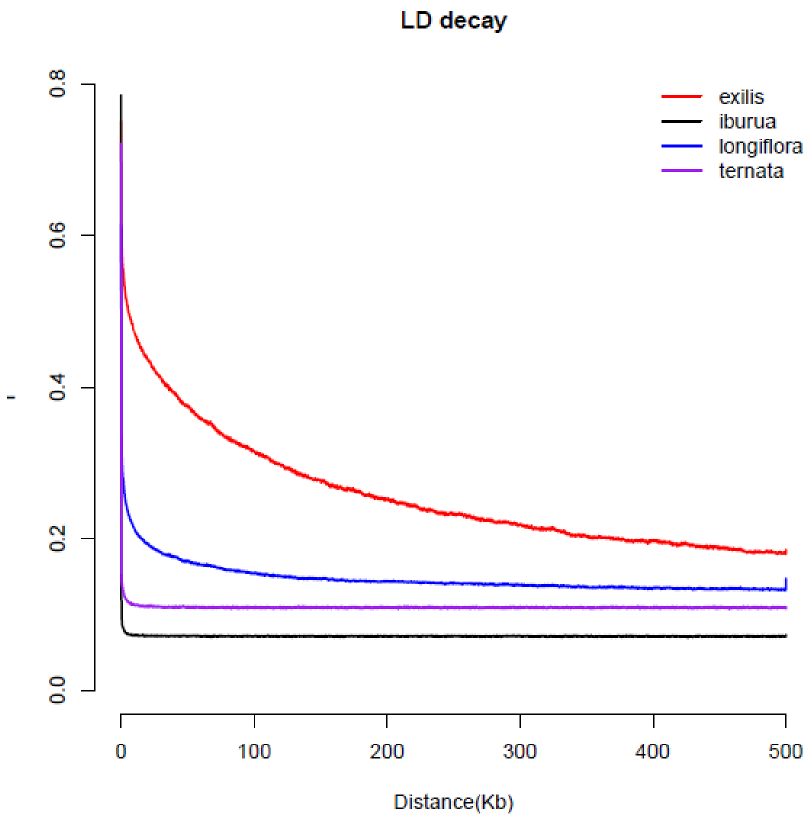

Supplementary Fig. 3. LD decay plot computed for each species across a 500kb distance.

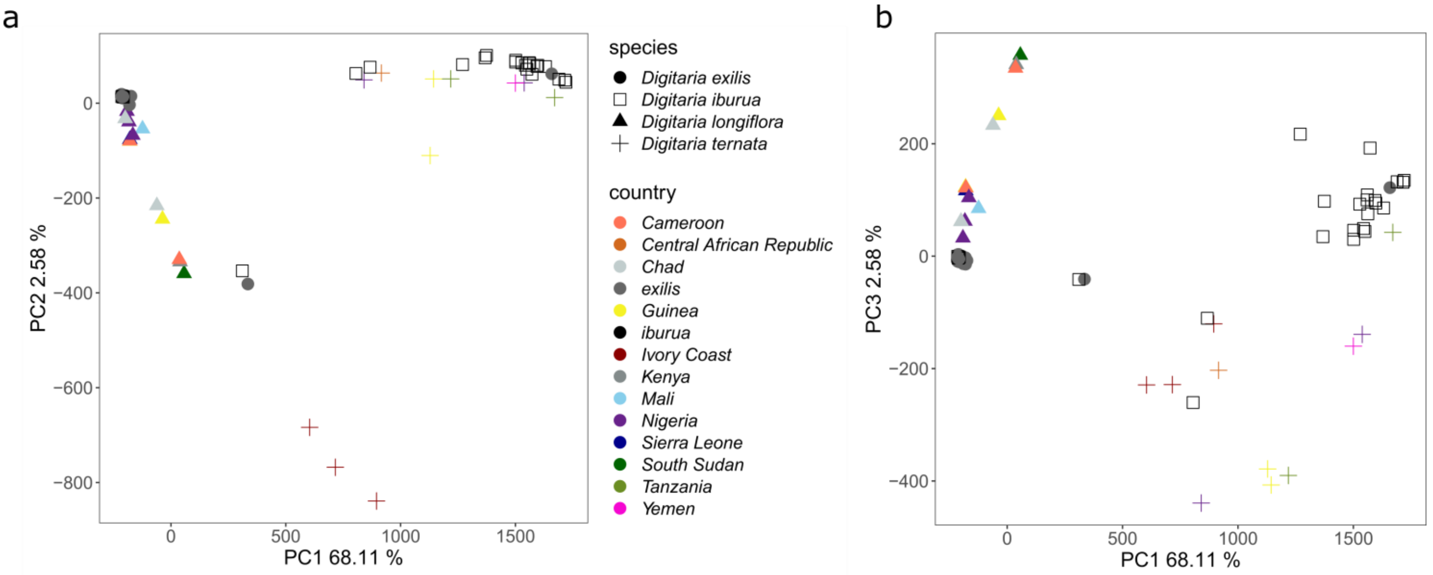

Supplementary Fig. 4. Principal component analysis of the genetic variation observed among the cultivated fonio millets and wild relatives (*n*=247 individuals, 438 883 SNPs with missing data <0.05). **a**. First and second axes displayed. **b**. First and third axes. Species are plotted with different shapes, with wild relatives coloured according to country of origin.

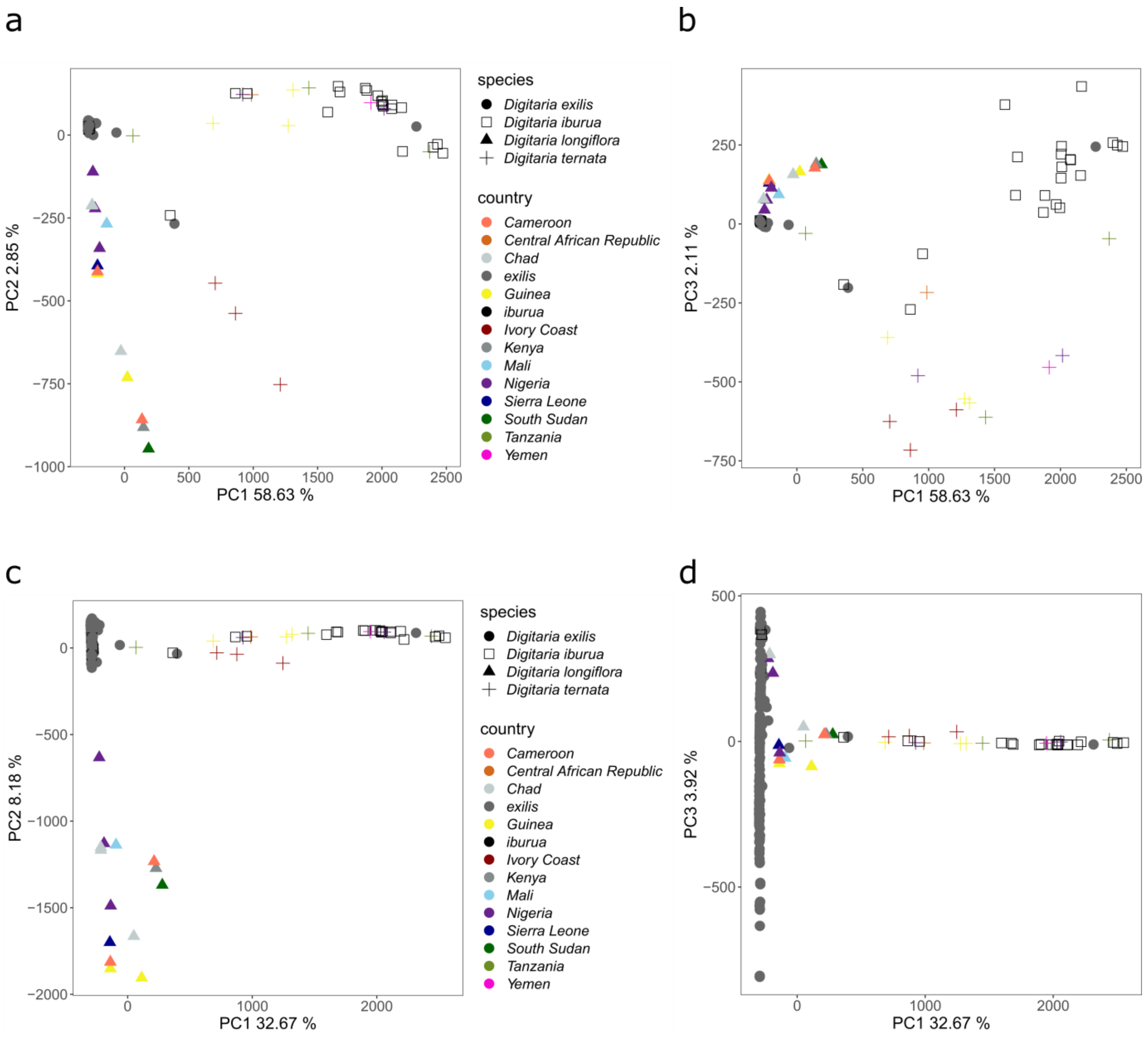

Supplementary Fig. 5. Principal component analysis of the genetic variation observed among the cultivated fonio millets and wild relatives for SNP datasets filtered with a 10% (top, *n*=250 individuals, 877 962 SNPs) or 20% (bottom, *n*=250 individuals, 1 731 962 SNPs) threshold for locus missingness. **a, c**: First and second axes displayed. **b, d**: First and third axes displayed. Species are plotted with different shapes, with wild relatives coloured according to country of origin.

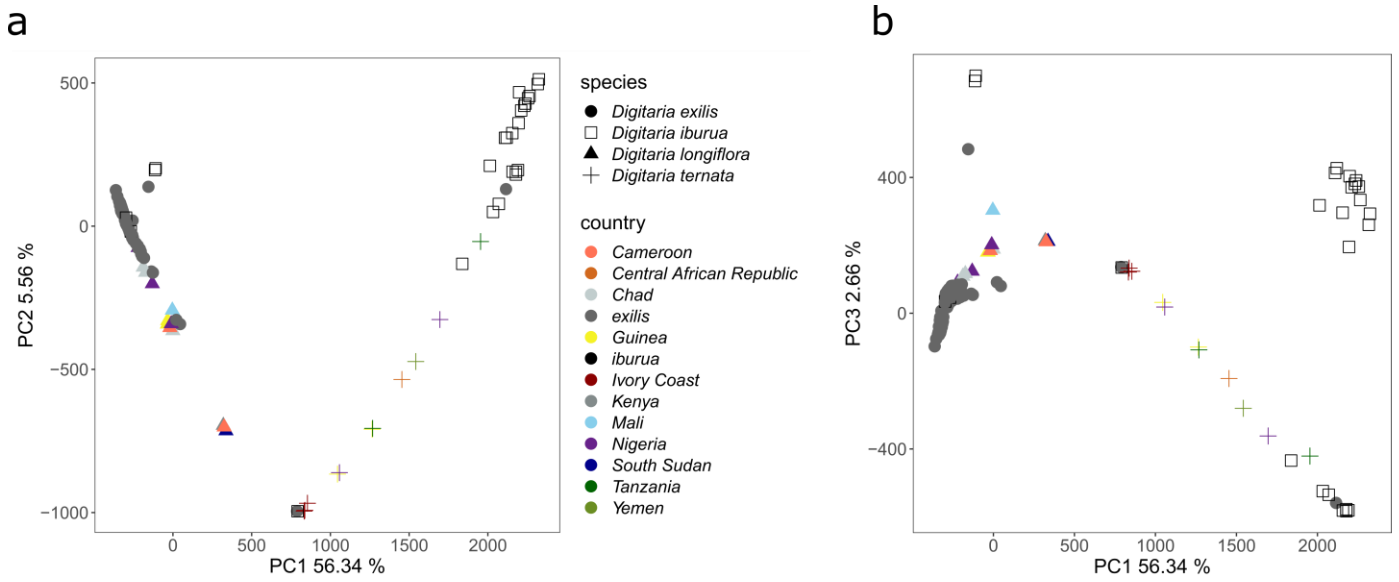

Supplementary Fig. 6. Principal component analysis of the genetic variation observed among the cultivated fonio millets and wild relatives, performed with the presence/absence table of 1,000,000 k-mers across 246 individuals. a. First and second axes displayed. **b**. First and third axes. Species are plotted with different shapes and colours correspond to country.

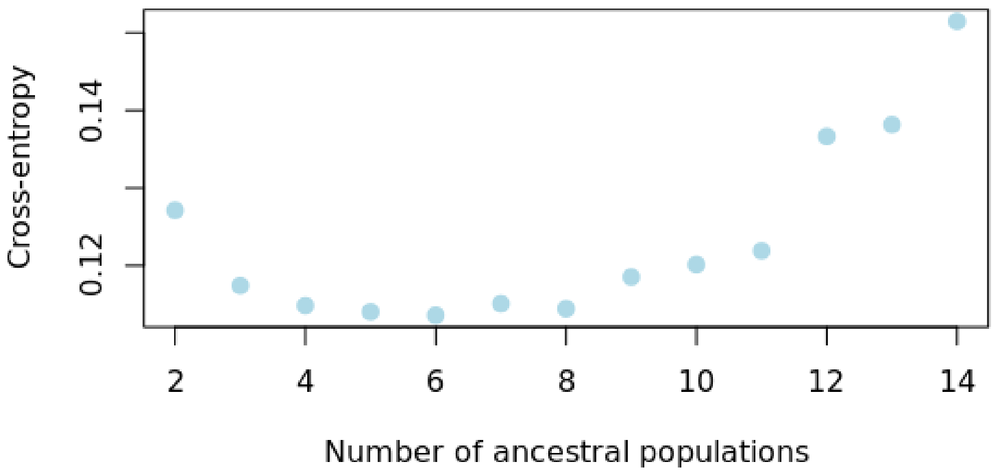

Supplementary Fig. 7. Mean values of the cross entropy estimated with the SNP dataset and computed across the 10 runs of sNMF performed for each K from K=2 to K=14.

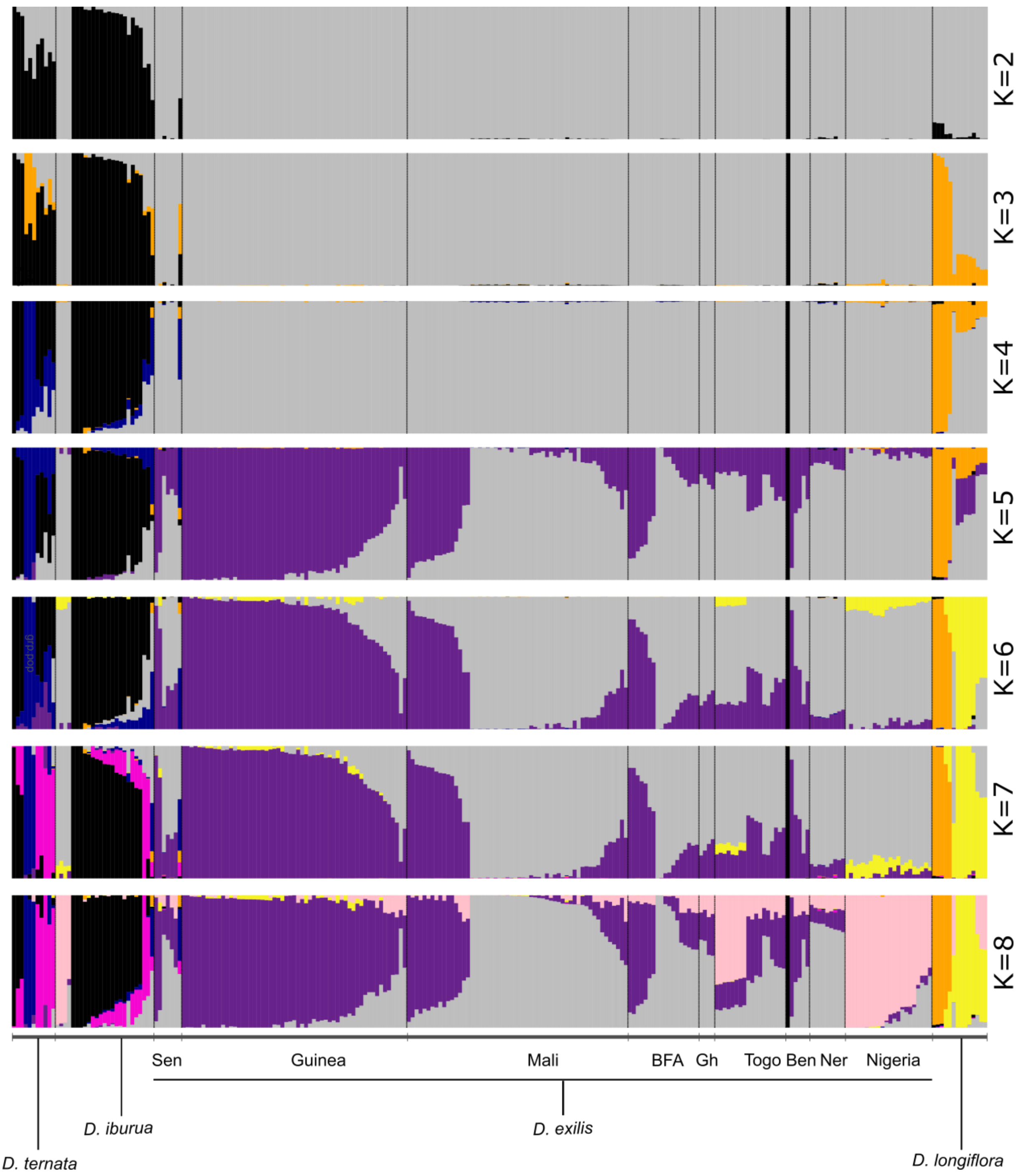

Supplementary Fig. 8. Population structure (from K = 2 to K = 8) of cultivated fonio millets (*D. exilis and D. iburua*) and wild relatives (*D. longiflora, D. ternata*) accessions estimated with sNMF using the 438 883 SNPs filtered for locus missingness and MAF at 0.05. Each individual is represented by a vertical bar, divided into K segments representing the proportion of genetic ancestry from the K clusters.

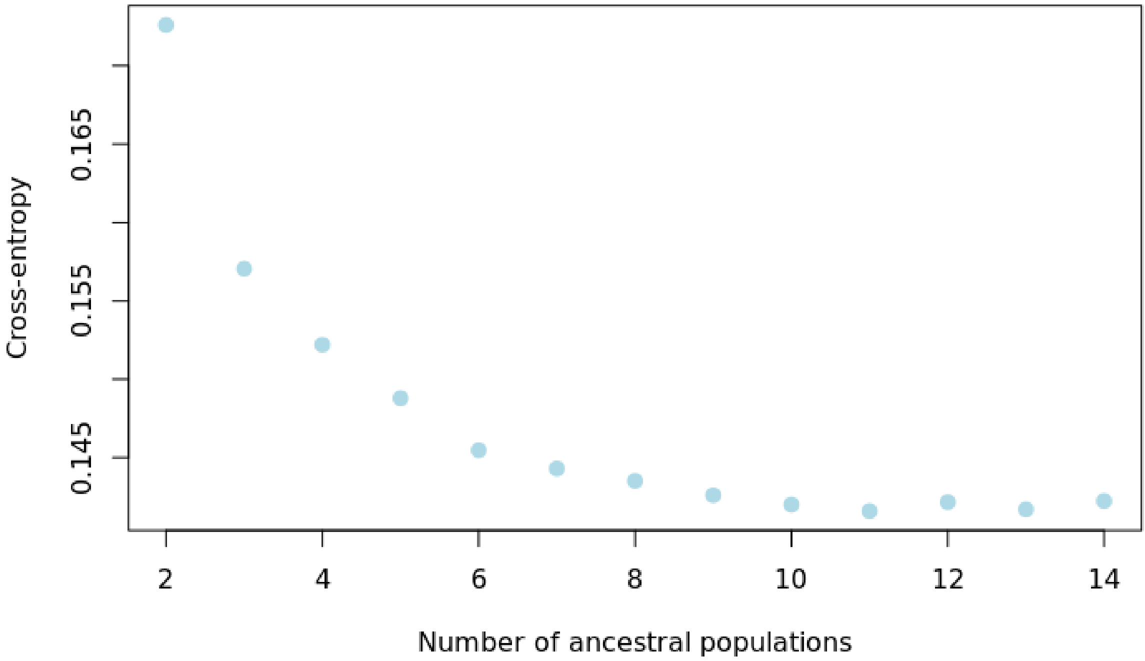

Supplementary Fig. 9. **Mean values of the cross entropy estimated with the k-mer dataset and computed across the 10 runs of sNMF performed for each K from K=2 to K=14.**

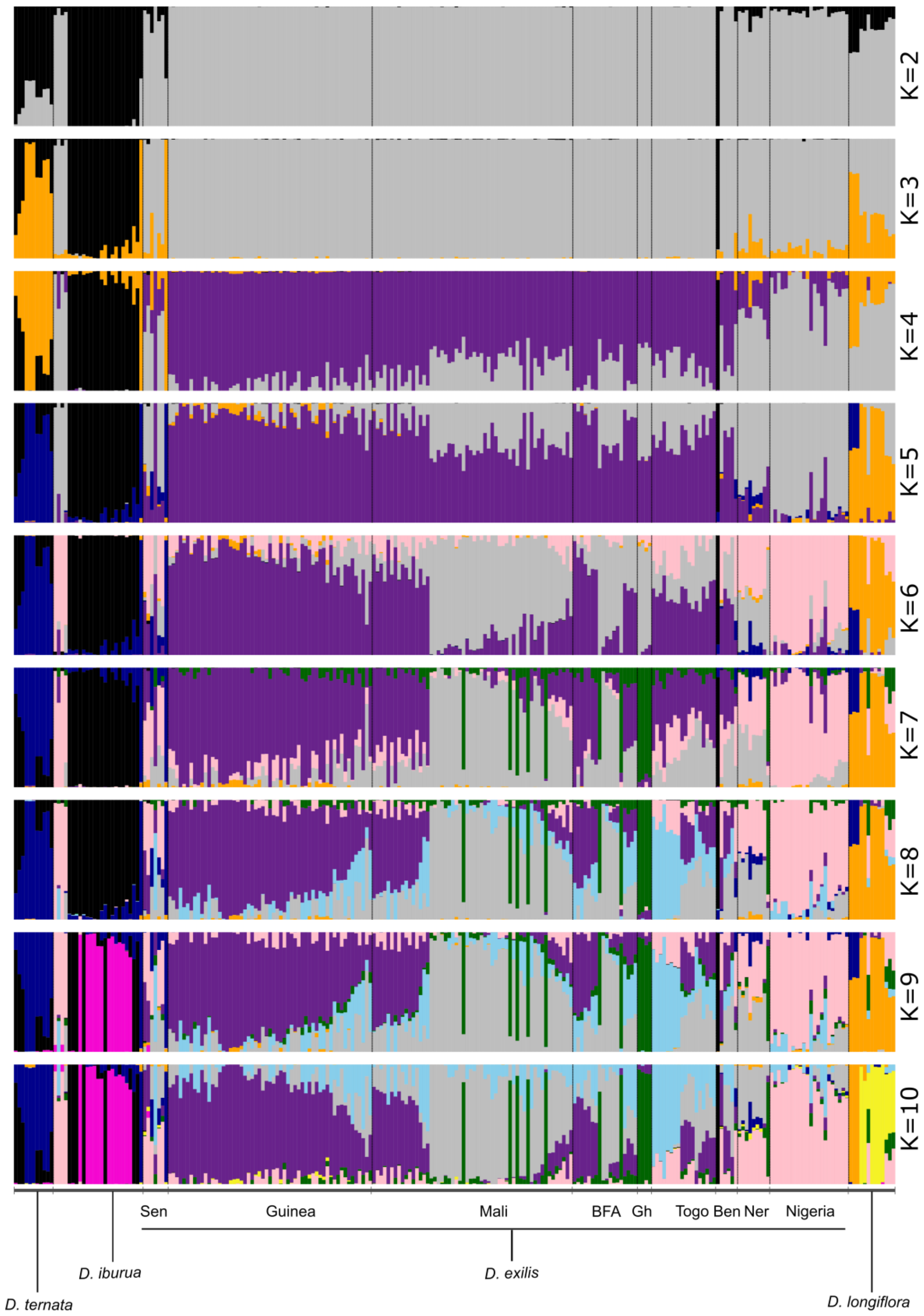

Supplementary Fig. 10. **Population structure (from K = 2 to K = 10) of cultivated fonio millets (*D. exilis* and *D. iburua*) and wild relatives (*D. longiflora*, *D. ternata*) accessions estimated with sNMF and using the presence/absence table of 1,000,000 k-mers among 246 individuals.** Each individual is represented by a vertical bar, divided into K segments representing the proportion of genetic ancestry from the K clusters.

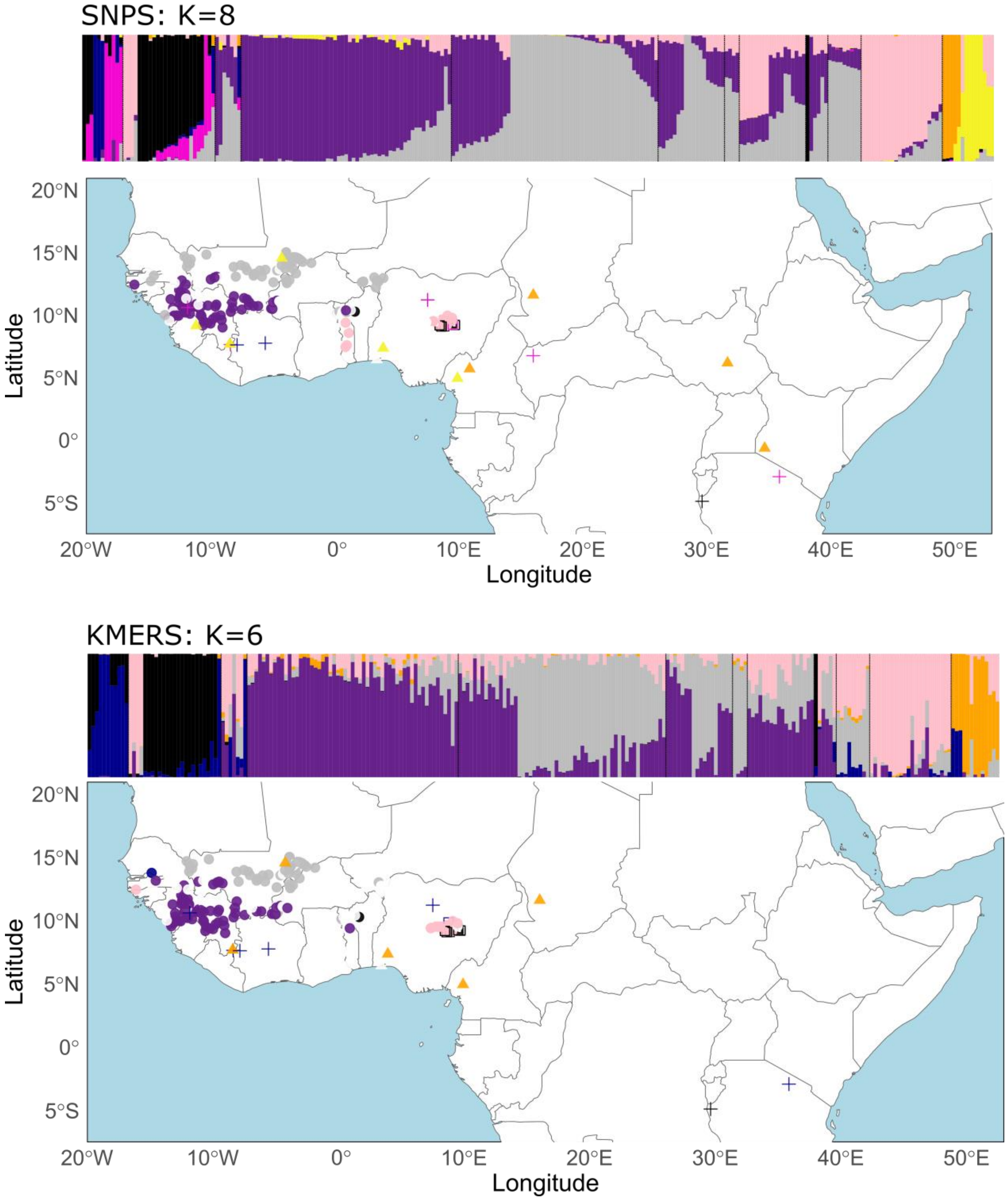

Supplementary Fig. 11. Population structure associated with the map of corresponding geo-referenced individuals for the SNP (top, 247 individuals) and k-mer (bottom, 246 individuals) datasets. Accessions are coloured according to their inferred population with a membership coefficient > 0.65. Admixed individuals (membership coefficient < 0.65) are not coloured. Species are highlighted with different shapes.

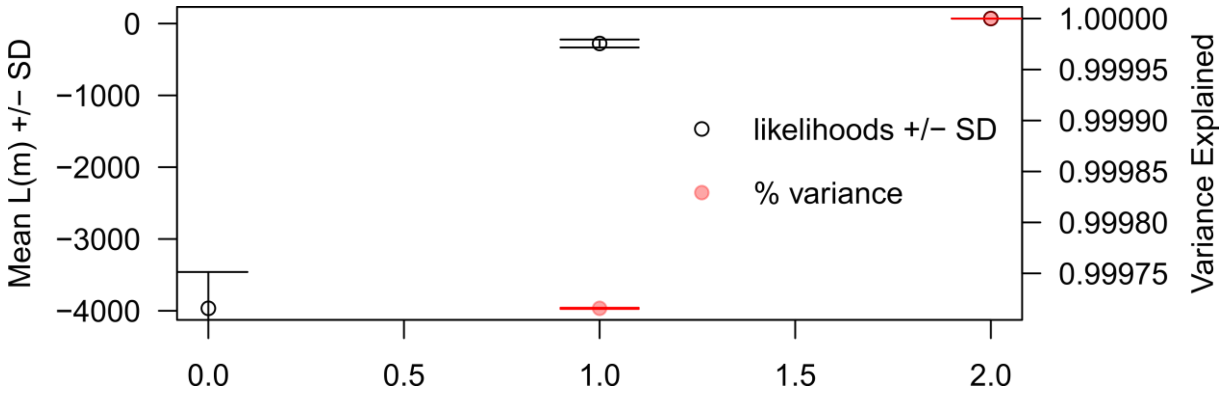

Supplementary Fig. 12. Plot of the mean log likelihood and percentage of variance explained estimated across 10 independent runs of TreeMix for different numbers of migration event m from m=0 to m=2. Four populations were considered, corresponding to the species identification in the passport data and the genetic results.

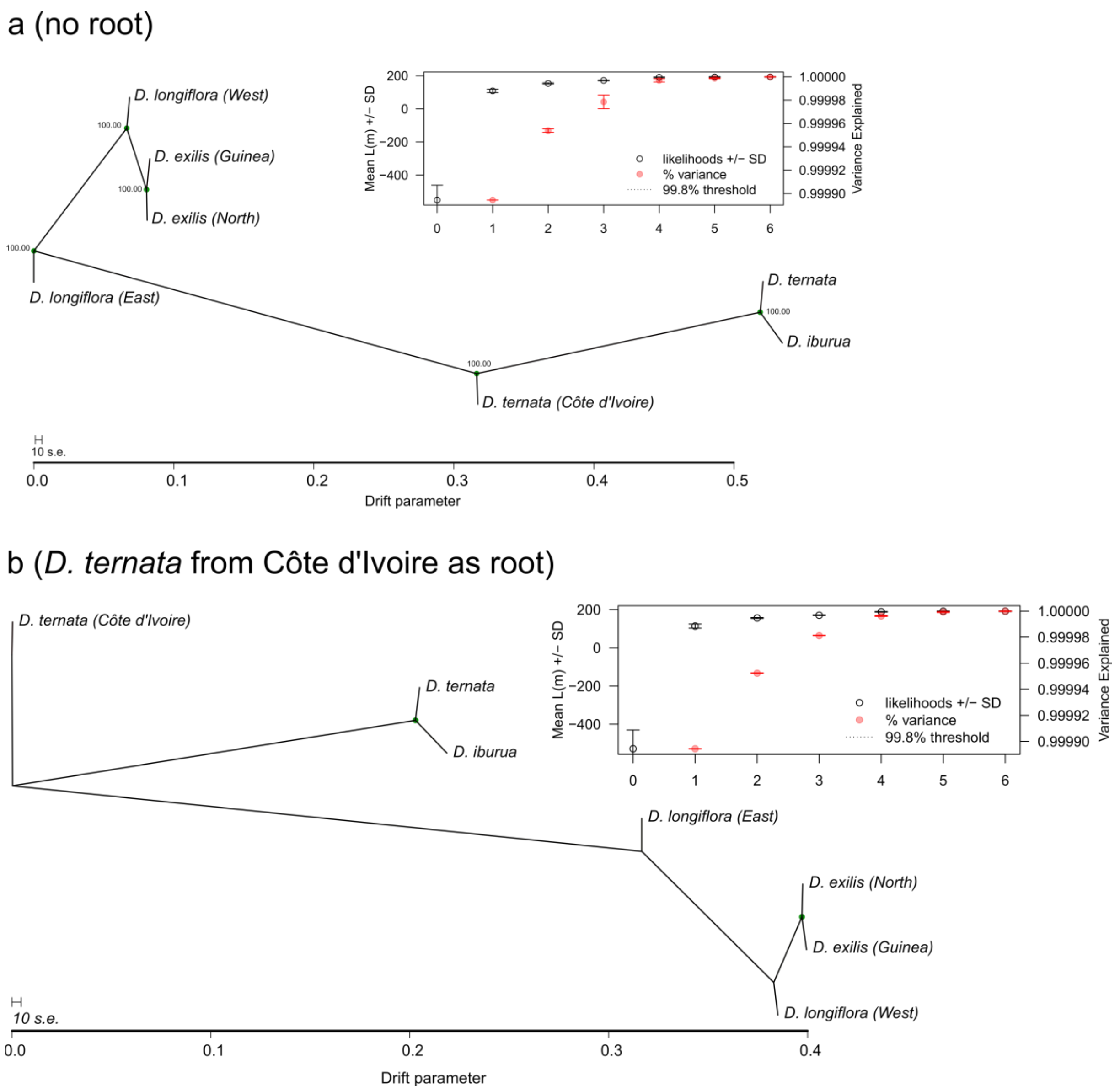

Supplementary Fig. 13. TreeMix models performed with seven populations corresponding to the species and genetic groups inferred with sNMF. The two groups of *D. exilis* correspond to the genetic clusters inferred at K=6 with sNMF. We also distinguished two geographic groups of *D. longiflora* and the *D. ternata* from Côte d'Ivoire. **a.** Model performed without specifying a root, with log-likelihood estimates when adding migration events from m=0 to m=6. **b.** Model performed without specifying a *D. ternata* (Côte d'Ivoire) as root, with log-likelihood estimates when adding migration events from m=0 to m=6.

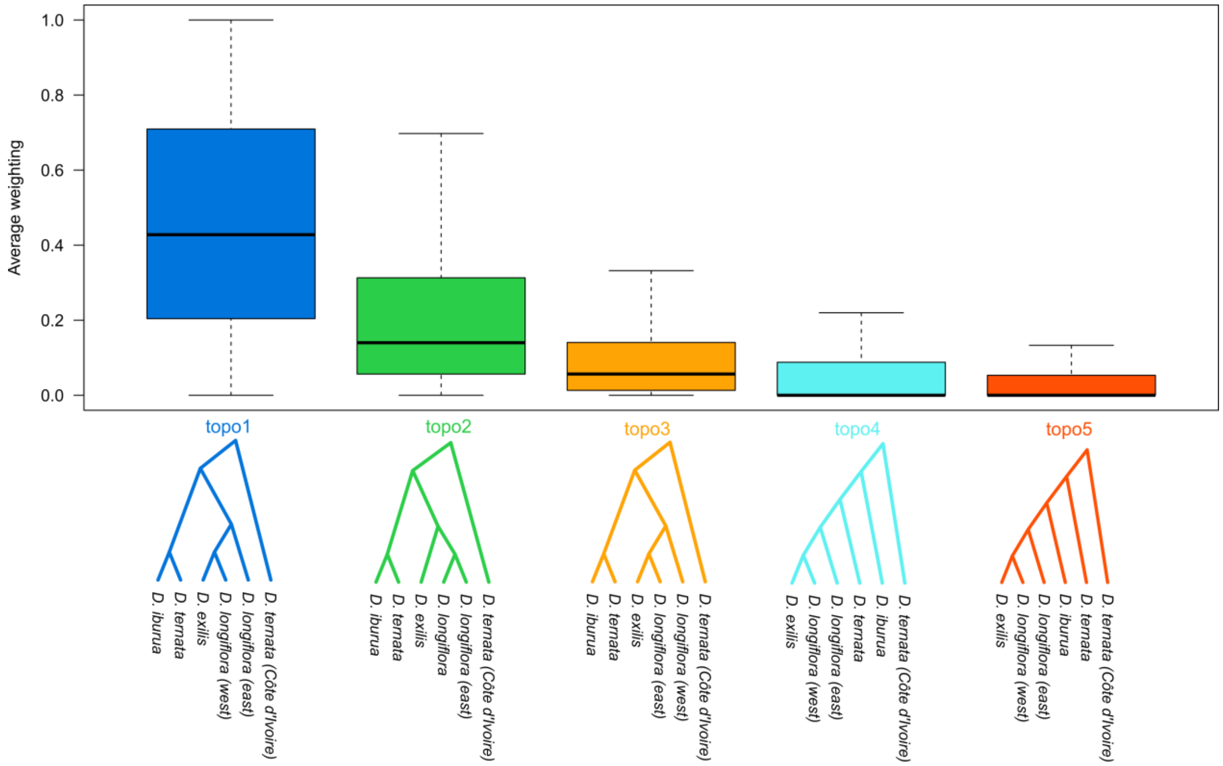

Supplementary Fig. 14. Topology weighting analysis of sliding window neighbour joining trees computed with six populations of cultivated fonio millets and wild relatives. See Supplementary Method 2. Barplots showing the distribution of the weights for each of the five most supported topologies are shown.

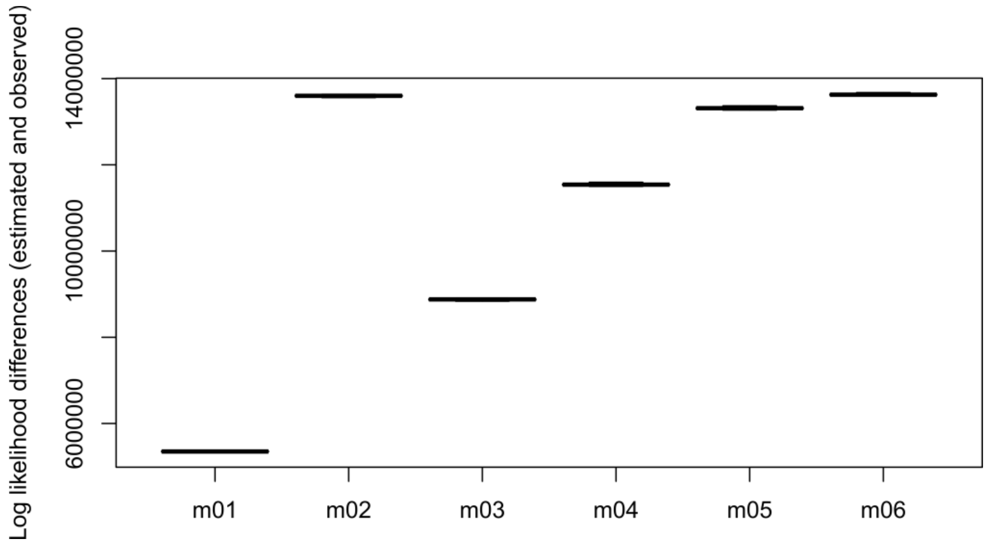

Supplementary Fig. 15. Fastsimcoal estimates of the maximum likelihood of each model simulated with 100 independent runs using as parameters the estimated point values derived from the best run previously obtained. The boxplots show the absolute differences between the observed and estimated log likelihoods of the six models simulated.

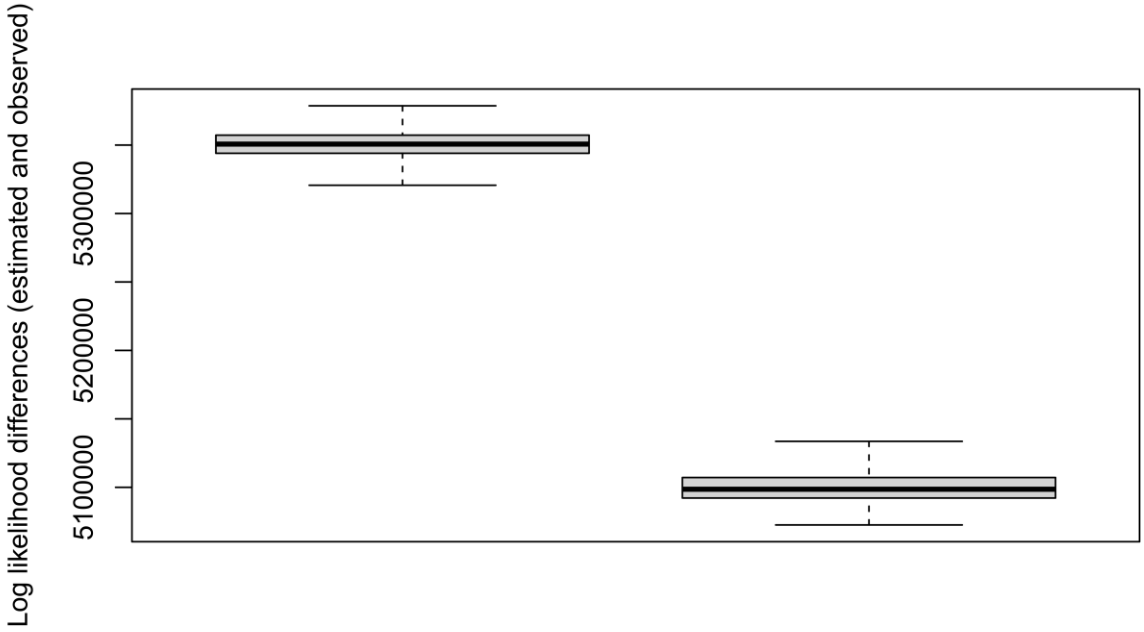

Supplementary Fig. 16. Fastsimcoal maximum likelihood estimates of models m01 and m01 assuming a population bottleneck for cultivated species following their divergence from their respective wild relative. The boxplots show the absolute differences between the observed and estimated log likelihoods for the two models, based on 100 independent runs using as parameters the estimated point values inferred by the previously obtained best run.

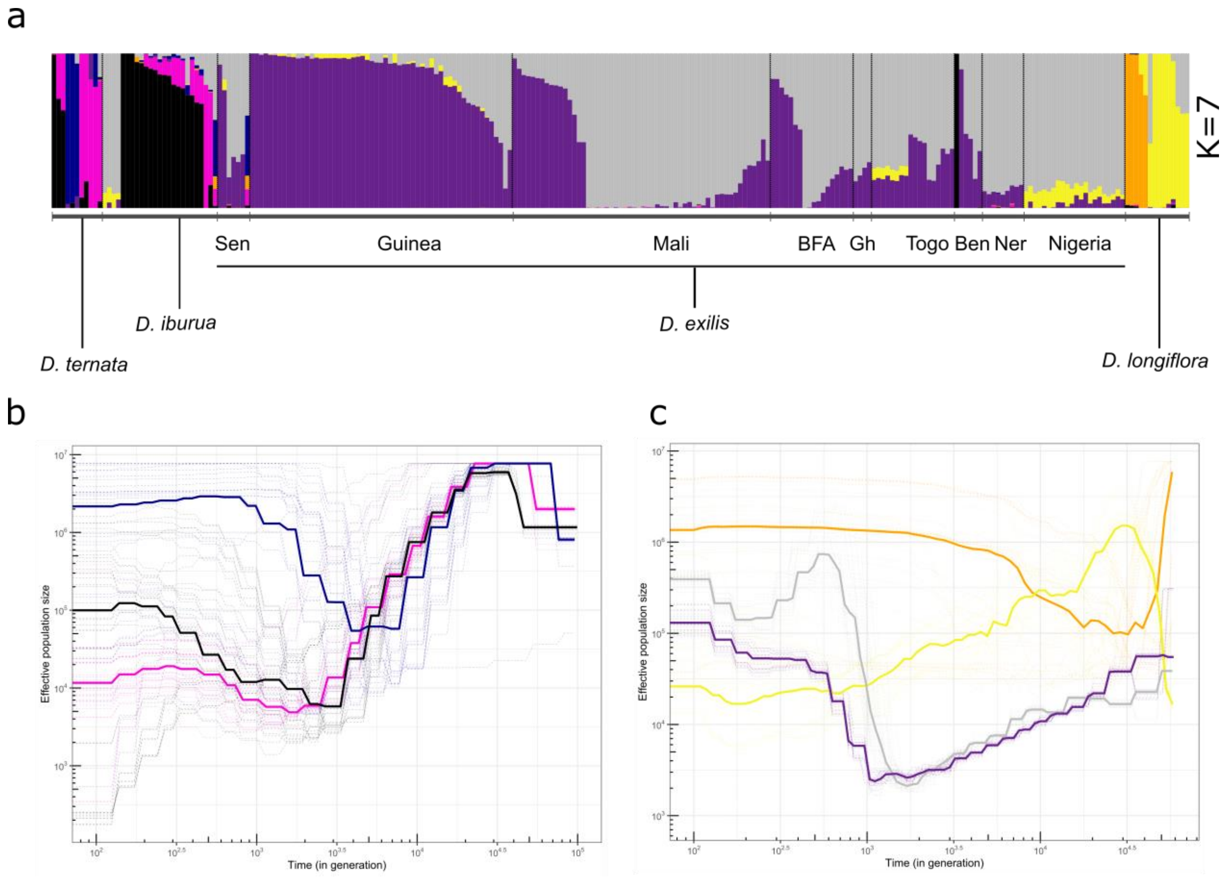

Supplementary Fig. 17. Inference of effective population size history of the genetic groups inferred for cultivated white and black fonio and wild relatives. Thin lines in smc++ plots represent the 30 independent runs of smc estimate and thick lines represent the median. **a.** Population structure at K=7 for the SNP dataset (438 883 SNPs, 247 individuals). **b.** smc++ plot for the two groups of *D. ternata* and the *D. iburua* cluster. **c.** smc++ plots for two groups of *D. longiflora* (east Africa and west Africa) and two groups of *D. exilis* (Nigerian and Guinean clusters).

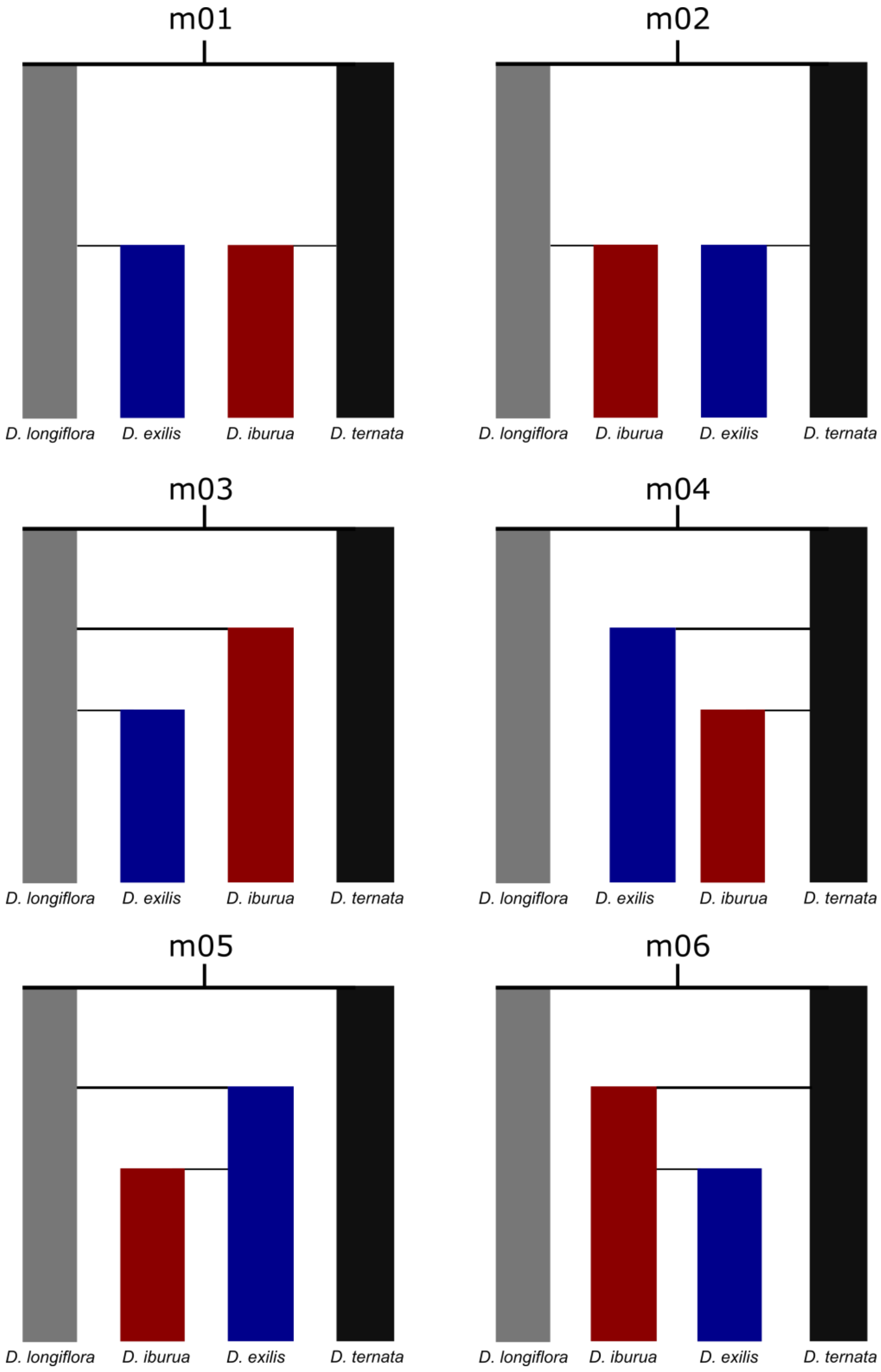

Supplementary Fig. 18. Scenarios of divergence tested with fastsimcoal v. 2.8. The scenarios differed according to the independence or not of the fonio millet crops and their pattern of divergence from wild relatives.
