# Supplementary material for: Independent domestication and cultivation histories of two West African indigenous fonio millet crops
Source: Nat Commun. 2025 Apr 30;16:4067. doi: 10.1038/s41467-025-59454-2 (PMC12044004; doi:10.1038/s41467-025-59454-2)
Supplement: Supplementary file 3 — Description of Additional Supplementary Files [file 41467_2025_59454_MOESM3_ESM.pdf]

**Description of Additional Supplementary Files**

**Supplementary Data 1.** Passport information of the 265 *Digitaria* accessions used in the study.

**Supplementary Data 2.** Mapping statistics of the 265 *Digitaria* accessions mapped against the *D. exilis* reference genome.

**Supplementary Data 3.** Proportion of missing data across individuals considering the SNP dataset filtered for a locus missing rate of 0.05 (1,910,119 SNPs).
