# Supplementary material for: Independent domestication and cultivation histories of two West African indigenous fonio millet crops
Source: Nat Commun. 2025 Apr 30;16:4067. doi: 10.1038/s41467-025-59454-2 (PMC12044004; doi:10.1038/s41467-025-59454-2)
Supplement: Supplementary file 7 — Reporting Summary [file 41467_2025_59454_MOESM7_ESM.pdf]

nature portfolio

Corresponding author(s): KACZMAREK T

Last updated by author(s): Apr 7, 2025

Reporting Summary

Nature Portfolio wishes to improve the reproducibility of the work that we publish. This form provides structure for consistency and transparency in reporting. For further information on Nature Portfolio policies, see our Editorial Policies and the Editorial Policy Checklist.

Statistics

For all statistical analyses, confirm that the following items are present in the figure legend, table legend, main text, or Methods section.

n/a	Confirmed	
☐	☒	The exact sample size (*n*) for each experimental group/condition, given as a discrete number and unit of measurement
☐	☒	A statement on whether measurements were taken from distinct samples or whether the same sample was measured repeatedly
☐	☒	The statistical test(s) used AND whether they are one- or two-sided<br>*Only common tests should be described solely by name; describe more complex techniques in the Methods section.*
☐	☒	A description of all covariates tested
☐	☒	A description of any assumptions or corrections, such as tests of normality and adjustment for multiple comparisons
☐	☒	A full description of the statistical parameters including central tendency (e.g. means) or other basic estimates (e.g. regression coefficient) AND variation (e.g. standard deviation) or associated estimates of uncertainty (e.g. confidence intervals)
☐	☒	For null hypothesis testing, the test statistic (e.g. *F*, *t*, *r*) with confidence intervals, effect sizes, degrees of freedom and *P* value noted<br>*Give P values as exact values whenever suitable.*
☒	☐	For Bayesian analysis, information on the choice of priors and Markov chain Monte Carlo settings
☒	☐	For hierarchical and complex designs, identification of the appropriate level for tests and full reporting of outcomes
☒	☐	Estimates of effect sizes (e.g. Cohen's *d*, Pearson's *r*), indicating how they were calculated

Our web collection on statistics for biologists contains articles on many of the points above.

Software and code

Policy information about availability of computer code

Data collection

Raw reads from 171 Digitaria exilis and Digitaria longiflora were downloaded from the European Nucleotide Archive (EBI-ENA) repository under the project number PRJEB36539. Sequences were retrieved using the enaGroupGet.py script from the enaBrowserTools github repository (https://github.com/enasequence/enaBrowserTools/tree/master).
The D. exilis reference genome was obtained from the DRYAD database at [https://doi.org/10.5061/dryad.2v6wwpzj0].
A total of 94 new genomes were generated in this study, and were made available in the EBI-ENA repository under the project number PRJEB80862.

Data analysis

The scripts used to perform the analyses of the sutdy, using the software listed below, are available at [https://gitlab.cirad.fr/agap/fonio/bwfonio].
FastQC v.0.11.9 was used to to analyze raw sequence reads.
Cutadapt v. 3.1 was used to trim the reads.
bwa-mem2 v.2.2.1 was used to map the raw reads to the D. exilis reference genome.
samtools v.1.14 was used for sorting and indexing reads.
GATK v.4.2.6.1 was used for SNP and genotype calling, and SNP filtering.
vcftools v.0.1.16 was used for SNP filtering and to produce missingness statistics.
The iKISS pipeline was used for the kmer analyses (https://forge.ird.fr/diade/iKISS).
egglib v.3.2.1 was used to compute genomic diversity statistics.
PopLDdecay v.3.42 used for LD decay estimation.
Treemix v.1.13 was used to infer tree topology and migration between populations. We adapted a pipeline from [https://github.com/carolindahms/TreeMix].

The twisst pipeline (https://github.com/simonhmartin/twisst) for topology weightying analyses.
smc++ v.1.15.4 to infer past changes in effective population size [https://github.com/popgenmethods/smcpp].
bcftools v.1.16, bedtools v.2.30.0 and msmc-tools (https://github.com/stschiff/msmc-tools) to produce smc++ input files.
fastsimcoal v.2.8 was used to test evolutionary scenarios and to infer demographic parameters.
ANGSD v.0.940 was used to compute site frequency spectrum.
R packages used:
ggplot2 v.3.4.2 for plotting and visualisation LEA v.3.9.5 for PCA and structure analyses; pophelper v.2.3.1 to display barplots of ancestry coefficients; optM v0.1.6 to determine optimal number of migration events inferred with Treemix v.1.13; ape v.5.7-1.

For manuscripts utilizing custom algorithms or software that are central to the research but not yet described in published literature, software must be made available to editors and reviewers. We strongly encourage code deposition in a community repository (e.g. GitHub). See the Nature Portfolio guidelines for submitting code & software for further information.

Data

Policy information about availability of data

All manuscripts must include a data availability statement. This statement should provide the following information, where applicable:

- Accession codes, unique identifiers, or web links for publicly available datasets
- A description of any restrictions on data availability
- For clinical datasets or third party data, please ensure that the statement adheres to our policy

Raw reads from 171 Digitaria exilis and Digitaria longiflora were downloaded from the European Nucleotide Archive (EBI-ENA) repository under the project number PRJEB36539.
The D. exilis reference genome was obtained from the DRYAD database at [https://doi.org/10.5061/dryad.2v6wwpzj0].
The raw sequencing data newly generated (94 new genomes) are available on EBI-ENA under the study number PRJEB80862.
The VCF and passport files of the 247 accessions are available at https://doi.org/10.18167/DVN1/OYTQO6.

Research involving human participants, their data, or biological material

Policy information about studies with human participants or human data. See also policy information about sex, gender (identity/presentation), and sexual orientation and race, ethnicity and racism.

Reporting on sex and gender	*Use the terms sex (biological attribute) and gender (shaped by social and cultural circumstances) carefully in order to avoid confusing both terms. Indicate if findings apply to only one sex or gender; describe whether sex and gender were considered in study design; whether sex and/or gender was determined based on self-reporting or assigned and methods used. Provide in the source data disaggregated sex and gender data, where this information has been collected, and if consent has been obtained for sharing of individual-level data; provide overall numbers in this Reporting Summary. Please state if this information has not been collected. Report sex- and gender-based analyses where performed, justify reasons for lack of sex- and gender-based analysis.*
Reporting on race, ethnicity, or other socially relevant groupings	*Please specify the socially constructed or socially relevant categorization variable(s) used in your manuscript and explain why they were used. Please note that such variables should not be used as proxies for other socially constructed/relevant variables (for example, race or ethnicity should not be used as a proxy for socioeconomic status). Provide clear definitions of the relevant terms used, how they were provided (by the participants/respondents, the researchers, or third parties), and the method(s) used to classify people into the different categories (e.g. self-report, census or administrative data, social media data, etc.) Please provide details about how you controlled for confounding variables in your analyses.*
Population characteristics	*Describe the covariate-relevant population characteristics of the human research participants (e.g. age, genotypic information, past and current diagnosis and treatment categories). If you filled out the behavioural & social sciences study design questions and have nothing to add here, write "See above."*
Recruitment	*Describe how participants were recruited. Outline any potential self-selection bias or other biases that may be present and how these are likely to impact results.*
Ethics oversight	*Identify the organization(s) that approved the study protocol.*

Note that full information on the approval of the study protocol must also be provided in the manuscript.

Field-specific reporting

Please select the one below that is the best fit for your research. If you are not sure, read the appropriate sections before making your selection.

☐ Life sciences ☐ Behavioural & social sciences ☒ Ecological, evolutionary & environmental sciences

For a reference copy of the document with all sections, see nature.com/documents/nr-reporting-summary-flat.pdf

Ecological, evolutionary & environmental sciences study design

All studies must disclose on these points even when the disclosure is negative.

Study description	We analyzed a large collection of fonio milet genomic resources comprising a total of 265 accessions, with the two cultivated species (D. exilis, D. iburua) and their close wild relatives (D. longiflora, D. ternata). We used different complementary methods and genomic approaches to unravel the domestication history of these fonio millets, which is key for unlocking the potential of these native species while preserving the diversity necessary for future adaptations.
Research sample	The research sample consists of 265 accessions of four species (D. exilis, D. iburua, D. longiflora, D. ternata). Of these accessions, 94 are new sequences and 171 were retrieved from a previous study.<br>For white fonio (D. exilis), we used 203 samples.<br>For black fonio (D. iburua), we used 26 samples.<br>For the wild species D. longiflora, we used 14 samples.<br>For the wild species D. ternata, we used 22 samples collected at the National Museum of Natural History in Paris (France) and in CIRAD herbariums.
Sampling strategy	For white and black fonio, the sampling strategy was defined to fully represent their geographical distribution. For D. exilis, accessions were selected to complement the previously available dataset. For D. iburua, the first whole genome sequences were obtained in this study. The samples thus represent a comprehensive representation of fonio millet diversity grown in family farming systems across West Africa.<br>We used wild relatives distributed throughout Africa, especially West Africa.
Data collection	For the 94 new sequences produced, accessions of cultivated species are conserved in national collections, and duplicates covered by the Material Transfer Agreement (MTA) are stored in the ARCAD gee bank (Montpellier, France). The D. ternata samples were collected from CIRAD and MNHN herbariums.<br>Marie Couderc and Sandrine Causse performed molecular analyses and constructed the sequencing libraries.<br>Sequencing was performed by the NOVOGEN company (China) using four Illumina sequencing lanes on a Illumina NovaSeq 6000 system.<br>Thomas Kaczmarek performed the bioinformatic analysis and SNP calling on the Core Cluster of the Institut Français de Bio-informatique (IFB).
Timing and spatial scale	The fonio millet seed collection was built up as part of several research projects from 1977 to 2021. Information for each sample sequenced is provided as supplementary data.<br>For D. exilis, samples represent nine countries of West Africa.<br>For D. iburua, samples originated from Nigeria, where it is almost exclusively cultivated.<br>The DNA sequencing data from the 94 new genomes were obtained in 2022.
Data exclusions	After mapping to the reference genome, we excluded accessions after with a mean depth < 1.<br>Only 250 accessions were considered for SNP and genotype calling.<br>We applied hard filters to the biallelic SNPs according to GATK guidelines by filtering SNPs with low (< 500) and high (>14,000) depth summed across all samples. Clusters of three SNPs within 10 bp were removed and we also excluded SNPs flagged with the conditions following the GATK best practices: QD < 2.0, FS > 60.0, SOR > 3.0, MQ < 40.0, MQRankSum < -12.50, ReadPosRankSum < -8.0.<br>We ended up with a filtered call set of 16,316,814 biallelic SNPs and 250 accessions.<br>For main genomic analyses, we filtered out SNPs that were missing in > 5% of the individuals and kept individuals with missing data < 0.40.<br>We ended up with 1,910,119 biallelic SNPs across 247 individuals.<br>For PCA and structure analyses, we filtered for minor allele frequency (MAF) at 0.05. Rare allele filtering is a common method for diversity and population structure analyses.<br>For fastsimcoal2 analyses, three D. ternata from Côte d'Ivoire, and five D. longiflora from East Africa were excluded as both were genetically far from the cultivated gene pools compared to the other wild individuals. We also considered a subsample of 21 D. exilis to be representative of the species diversity and similar in sample size to D. iburua (N=19).<br>For smc++, we used the msmc-tools (https://github.com/stschiff/msmc-tools) advices and pipeline to label positions as missing data (across all samples) corresponding to insufficiently covered regions.
Reproducibility	The Treemix results obtained are based on multiple steps of bootstrap replicates.<br>We performed 30 independent runs of smc++ and plotted the median to estimate the variation in effective population size.<br>The maximum likelihood estimates for the domestication scenario tested with fastsimcoal2 are based on 100 independent runs of 500,000 coalescent simulations, plus 100 new runs using the estimated point values of parameters inferred by the previously obtained best run.<br>Once we determined the most likely fonio millet domestication scenario, we estimated confidence intervals of the parameter estimates through a parametric bootstrap approach. Using the best set of parameters, we generated 500,000 SFS from 100 independent pseudo-observed datasets of 200,000 non-recombining DNA segments of 1,000 bp.
Randomization	We did not conduct phenotyping experiments that required randomisation of samples.<br>Allocation of samples into groups for population modelling (treemix, smc++, fastsimcoal2) or based on species delimitation and/or clusters inferred by structure analyses.
Blinding	Blinding was not relevant to our study as it involved resequencing data for population genomic analyses.

Did the study involve field work? ☐ Yes ☒ No

Reporting for specific materials, systems and methods

We require information from authors about some types of materials, experimental systems and methods used in many studies. Here, indicate whether each material, system or method listed is relevant to your study. If you are not sure if a list item applies to your research, read the appropriate section before selecting a response.

Materials & experimental systems

n/a	Involved in the study
☒	☐ Antibodies
☒	☐ Eukaryotic cell lines
☒	☐ Palaeontology and archaeology
☒	☐ Animals and other organisms
☒	☐ Clinical data
☒	☐ Dual use research of concern
☐	☒ Plants

Methods

n/a	Involved in the study
☒	☐ ChIP-seq
☒	☐ Flow cytometry
☒	☐ MRI-based neuroimaging

Plants

Seed stocks	White and black fonio accessions are conserved in national collections, and duplicates covered by the Material Transfer Agreement (MTA) are stored in the ARCAD gee bank (Montpellier, France).
Novel plant genotypes	not applicable
Authentication	not applicable
